# The Human Genetics of Dental Anomalies

**DOI:** 10.1055/s-0042-1743572

**Published:** 2022-02-25

**Authors:** Mahamad Irfanulla Khan, Nadeem Ahmed, Praveen Kumar Neela, Nayeem Unnisa

**Affiliations:** 1 Department of Orthodontics & Dentofacial Orthopedics, The Oxford Dental College, Bangalore, Karnataka, India; 2 General Dental Practitioner, Max Dental Specialties, Bangalore, Karnataka, India; 3 Department of Orthodontics & Dentofacial Orthopedics, Kamineni Institute of Dental Sciences, Narketpally, Andhra Pradesh, India; 4 General Dental Practitioner, The Dental Clinic, Bangalore, Karnataka, India

**Keywords:** tooth development, dental anomalies, genetics, genes, mutations

## Abstract

The development of tooth is a highly complex procedure and mastered by specific genetic programs. Genetic alterations, environmental factors, and developmental timing can disturb the execution of these programs, and result in various dental anomalies like hypodontia/oligodontia, and supernumerary teeth, which are commonly seen in our clinical practice. Advances in molecular research enabled the identification of various genes involved in the pathogenesis of dental anomalies. In the near future, it will help provide a more accurate diagnosis and biological-based treatment for these anomalies. In this article, we present the molecular phenomenon of tooth development and the genetics of various dental anomalies.

## Introduction

The tooth is a specialized organ of the maxillofacial skeleton and its development involves a series of lengthy and complex stages. 1 Tooth development is influenced by genetic and environmental factors. 2 Tooth number, position, structure, and shape are under the control of a complex system of certain genes whose modifications may result in dental anomalies. 3 Depending on the stage of tooth development where alteration takes place, different dental anomalies could occur in terms of number (anodontia, hypodontia, and hyperdontia), structure (amelogenesis imperfecta [AI], dentinogenesis imperfect, and dentin dysplasia), and shape (microdontia, macrodontia, and taurodontism). 4 These anomalies may be associated with the systemic disorder (syndromic) or isolated (nonsyndromic). Dental anomalies may create problems of aesthetics as well as speech, which requires a multidisciplinary approach for the treatment of these anomalies. 5 6

Advances in molecular genetics enabled us to identify the various genes involved in the pathogenesis of dental anomalies. This article describes the molecular phenomenon of tooth development and the genetics of various dental anomalies.

## Tooth Development and Genetic Basis of Dental Anomalies

In humans, the development of teeth involves several distinctive cellular and molecular interaction processes. The deciduous and permanent teeth develop from the oral ectoderm and the underlying neural mesenchymal cells, which have migrated from the cranial neural crest to the facial process. 7 During the 6th week of human development, a line of oral epithelium cells condenses to form the dental lamina, which develops several tooth buds, invading the underlying mesenchyme. These reciprocal interactions of epithelial cells and mesenchymal tissues modulate tooth development. 8 The center of the epithelial mass known as the enamel knot functions as an important signaling center for the regulation of tooth shape. 9

The earliest stages of human embryogenesis involve the formation of the head for which a specific group of cells with stem cell properties called cranial neural crest cells (CNCs) are of importance. Except for muscles that are formed by mesodermal cells, the CNC is responsible for the development of the entire head structures. 10 The CNC further specifies and is organized into particular elements like bones and teeth by way of a unique continuous “molecular dialogue” or interaction with the epithelium that covers the developing face and oral cavity. 11 The interaction of CNC with epithelium through this molecular dialogue involves certain proteins that are products of specific genes. These proteins command cells to either divide or die (apoptosis), migrate/proliferate, or differentiate into more distinct cell types such as osteoblasts, odontoblasts, and chondrocytes. 12 13

Orofacial and dental disorders occur as a result of mutations in the sequence of either a gene or a group of genes because of alterations to the expression or function of the encoded protein(s). 14 Apart from genetic mutations, environmental factors can affect the expression of genes or interfere with the normal function of their protein products. 15 The position, number, and shapes of different types of teeth are determined by more than 300 genes. 16 The homeobox (HOX) genes are the most commonly studied genes in relation to tooth development, and they are expressed in the migrating neural crest cells. Between 18 and 24 weeks of development, the HOX gene network appears to be active in human tooth germs. 17 The PAX, MSX, DLX, LHX, BARX, and RUNX2 are the key members of the HOX genes engaged in the development of the tooth. 18

The BMP (bone morphogenetic protein), FGF (fibroblast growth factor), TGFb (transforming growth factor b), EGF (epidermal growth factor), SHH (sonic hedgehog), and the WNT (wingless) families are the major signaling molecules involved in the regulation of tooth embryogenesis. 19 20 The Msx-2 gene expression was localized to enamel knot cells, and its expression during tooth development provided a molecular link between tooth initiation and shape. 21 Literature database has shown that the mutations in many of these genes cause dental anomalies in mice as well as in humans. 22 23

## Genes Involved In Dental Anomalies

### Tooth Number Anomalies

Tooth number anomalies are characterized by either the presence in excess or absence of teeth than the usual number of primary or permanent dentitions due to changes in the dental lamina or tooth germ during tooth development. 24

#### Tooth Agenesis

Tooth agenesis (OMIM # 106600) refers to the congenital absence of teeth and occurs as a consequence of disturbances in their initial tooth development stages of tooth formation and proliferation. Its prevalence is between 1.6 and 9.6% and is seen more in females than in males. The most common teeth that show tooth agenesis are the third molars (9–30%), followed by mandibular second premolars (3–4%) and then maxillary lateral incisors (2.2%) and maxillary second premolars. Except for maxillary lateral incisors, unilateral agenesis is more frequent than bilateral agenesis. 25

The etiology of tooth agenesis is multifactorial, which includes genetic, epigenetic, and environmental factors. 26 It may occur as an isolated (nonsyndromic) or as a part of syndromes (syndromic). Several environmental factors like irradiation, chemotherapeutic agents, and dioxin can arrest tooth development. But the genetic factor supersedes the environmental factor in causing tooth agenesis, which is supported by molecular studies of familial autosomal dominant tooth agenesis associated with mutations in genes expressed in early tooth development such as paired box 9 (PAX9), muscle segment homeobox 1 (MSX1), axis inhibitor 2 (AXIN2), and ectodysplasin A (EDA). Tooth agenesis occurs more frequently in monozygotic twins and it indicates a notable influence of genetic factors. 27 28

A literature search determined the most important candidate genes whose mutations are responsible for tooth agenesis include *MSX1* , *PAX9* , *EDA* , ectodysplasin A receptor ( *EDAR* ), *AXIN2* , and Wingless type 10A ( *WNT10A* ) genes. The genes involved in the development of various dental anomalies are presented in Table 1 .

**Table 1:** Genes involved in the development of dental anomalies in humans

Sl. no.	Dental anomaly	OMIM	Gene(s)	Studies
1	Tooth agenesis	106600	*MSX1* , *PAX9* , *AXIN2* , *WNT10A* , and *EDA*	Gerits et al, 27 Lidral and Reising, 29 Stockton et al, 32 Lammi et al, 39 Kantaputra and Sripathomsawat, 41 Song et al 42
2	Supernumerary teeth	187100	*RUNX2* , *APC*	Cohen, 51 Inchingolo et al 52
3	Amelogenesis imperfecta	104530	*AMELX* , *ENAM* , *MMP20* , *KLK4* , *DLX3*	Greene et al, 55 Ozdemir et al, 57 Kim et al, 58 Hart et al, 59 Dong et al 60
4	Dentinogenesis imperfecta	125490	*DSPP*	Lee et al, 66 Zhang et al 68
5	Taurodontism	272700	*DLX3* , *MMP2*	Whitehouse et al, 74 Andersson et al 75

### Nonsyndromic Tooth Agenesis

Isolated, nonsyndromic tooth agenesis can present as sporadic or familial and may be inherited as an autosomal dominant, recessive, or X-linked mode. It may be classified as hypodontia, oligodontia, or anodontia. The absence of one to six teeth excluding the third molars is termed hypodontia, and the absence of more than six teeth excluding the third molars is known as oligodontia (OMIM #604625). The congenital absence of all teeth is termed anodontia (OMIM #206780) and is usually associated with syndromes.

#### Muscle Segment Homeobox 1 (MSX1)

Human MSX1 gene located at 4p16.1 spans around 4.05 kb and consists of two exons that encode a homeodomain—a 297 amino acid protein and 1 intron. 29 It plays a vital role during the development of teeth and craniofacial structures. 30 Mutations in the MSX1 gene mapped to 4p16.2 showed a second premolar and a third molar hypodontia. 31

#### The Paired Box 9 (PAX9)

PAX9 gene is a member of the paired box (PAX) family and is located at 14q13.3. They encode proteins that share a 128-amino acid DNA binding domain and are important regulators of numerous developmental processes. 32 33 Pax9 plays an important role during tooth development, and high levels of Pax9 expression are maintained throughout the initiation, bud, cap, and bell stages of tooth development. 34 35 Nieminen et al and Klein et al reported mutations of the PAX9 gene led to nonsyndromic oligodontia of the molar teeth. 36 37 Several studies indicated an important partnership between the thePax9 paired domain protein and the Msx1 homeoprotein in regulating gene expression in dental mesenchymal tissue. In humans, a heterozygous mutation in either PAX9 or MSX1 causes tooth agenesis of molar and premolar teeth, respectively. 38

#### The Axis inhibitor 2 (AXIN2)

AXIN2 gene is located at 17q24.1 and encodes the Axin2 protein that plays an important role in regulating the stability of β-catenin, which is involved in the Wnt signaling pathway (wingless). AXIN2 mutations result in hypodontia and oligodontia associated with colorectal cancer. 39 It was found that the oligodontia caused by the AXIN2 gene was more severe than that described for mutations in MSX1 and PAX9 genes; there were more missing molars, premolars, upper lateral incisors, and lower incisors. 40

#### Wingless-type 10A (WNT10A)

*WNT10A* gene, located at 2q35, contains 4 exons and belongs to the WNT gene family encoding the expression of signaling proteins on the cell surface and is associated with several syndromes (ectodermal dysplasia), but also nonsyndromic hypodontia. 41

#### Syndromic Tooth Agenesis

Mutations in the genes that are involved in tooth development as well as development of other organs result in tooth agenesis, which is associated with many syndromes. Syndromes associated with tooth agenesis are ectodermal dysplasia, Rieger's syndrome, and Witkop's tooth and nail syndrome. 42 In ectodermal dysplasia, mutations in EDA and gap junction protein Beta 6 (GJB6) were observed. The MSX1 homeobox gene are responsible for Witkop's tooth and nail syndrome, and Rieger's syndrome is a result of mutations in paired-like homeodomain transcription factor 2 (PITX2) gene, which is involved in tooth development. 43

Mutations in the EDA gene mapped at Xq13.1 showed an X-linked hypohidrotic ectodermal dysplasia (HED), a rare disease characterized by hypoplasia or absence of sweat glands, dry skin, sparse hair, and pronounced oligodontia. 44 45

#### Supernumerary Teeth

Supernumerary teeth or hyperdontia (OMIM #187100) are characterized by an excess in the number of teeth from usual for any given region of the dental arch. They may occur unilaterally or bilaterally, single or in multiples in any region of dental arches. 46 Both primary and permanent dentitions are affected, but most commonly seen in permanent dentition with the prevalence rate of 0.3 to 3.0%. Patients with supernumerary primary teeth may have 30 to 50% chances of these being followed by supernumerary permanent teeth. They are more frequently present in males than in females. Premaxilla is the most common site of its occurrence with mesiodense. 47 48

Different explanations ranging from the dichotomy of tooth germs and hyperactivity of dental lamina to remnants of epithelial cells were proposed, but the etiology remains unclear. Although genetic influences are reported in relation to its occurrence, environmental factors were also responsible. Supernumerary teeth may be transmitted through autosomal dominant or autosomal recessive traits with incomplete penetrance or may be associated with an X chromosome. In most cases, supernumerary teeth have been reported in patients with syndromes such as cleidocranial dysplasia, Ehlers–Danlos syndrome type III, Ellis–Van Creveld syndrome, Gardner's syndrome, Goldenhar's syndrome, Hallermann–Streiff syndrome, orofaciodigital syndrome type I, Nance–Horan syndrome, and cleft lip and/or palate. 49 50

Genetic studies found heterozygous mutations of the runt-related transcription factor 2 ( *RUNX2* ) gene, which is located at 6p21, to be responsible for the development of cleidocranial dysplasia. 51 A mutation in the APC gene located on chromosome 5q22.2 is found to be a causative factor for Gardner's syndrome. It is also proposed that inactivation of APC or forced activation of Wnt/β (catenin signaling) results in multiple supernumerary teeth formation in both humans and mice, but key genes in these pathways are not very clear. 52

### Structural Anomalies

#### Amelogenesis Imperfecta

AI (OMIM # 104530) refers to a rare, genetically, and clinically heterogeneous group of inherited disorders characterized by abnormal enamel formation qualitatively or quantitatively. It can present as locally deficient enamel as hypoplasia or aplasia, which is complete enamel absence. Clinically a patient with AI shows yellow to brown-colored teeth with or without opacities, microdontia to normal size depending on enamel thickness. 53 The prevalence of AI ranges from 1 in 700 to 1 in 4,000 in different populations. It may present as autosomal dominant, autosomal recessive, or X linked. Usually, AI is a nonsyndromic enamel defect, but other dental anomalies may be associated with it like pulp calcification, taurodontism, delayed eruption, and gingival overgrowth. The AI phenotype is broadly categorized into hypoplastic, hypomaturation, and hypocalcified. 54

The etiology is poorly understood and controversial be it a local mechanism, environmental aspect, or genetic mutations. Present literature reports that the amelogenin (AMELX), 55 enamelin (ENAM), 56 57 matrix metalloproteinase 20 (MMP20), 58 and kallikrein 4 (KLK4) 59 genes are involved in the development of AI. But it is not clear that all AI causing genes have been identified. The distal-less homeobox 3 (DLX3) gene causes AI as a part of trichodento-osseous (TDO) syndrome. 60 If the hair and bone abnormalities in TDO are not noticeable, the condition is designated AI hypoplastic hypomaturation with taurodontism (AIHHT). 61 62

#### Dentinogenesis Imperfecta

Dentinogenesis imperfecta (DI; OMIM # 125490) is a hereditary developmental defect of dentin formation that results in the appearance of opalescent teeth. The teeth usually appear blue-gray or yellow brown in color. 63 It is generally inherited in simple autosomal dominant mode with high penetrance and low mutation rates. Both the primary and permanent dentition is affected by DI. The overlying enamel is normal in structure but breaks easily due to poor dentin exposing it to the oral environment, which results in weaker teeth prone to recurrent bacterial infections. 64 65

Although the classification provided by Witkop and Shields are well accepted, they are not satisfactory. Genetic studies on DI type 1 have confirmed that osteogenesis imperfecta is a separate entity from DI and only a single genetic mutation of the dentin sialophosphoprotein (DSPP) gene located at chromosome 4q21.3 is responsible for causing DI type 2 and DI type 3. 66 67 The gene product is a precursor protein that is halved into two dentin-specific proteins: dentin sialoprotein (DSP) and dentin phosphoprotein (DPP). The genetic basis for this heterogeneity is not known. Osteogenesis imperfecta results from mutations in gene encoding type 1 collagens—collagen 1A1 (COL1A1) and collagen 1A2 (COL1A2)—whereas DI is associated with DSPP gene mutation. 68 69 An early diagnosis of DI is important in preventing further attrition of teeth as well as reduction in the vertical dimension of occlusion, so as to preserve function, aesthetics, and normal growth.

### Genetic Defects Manifested Late in Tooth Development

#### Taurodontism

Taurodontism (OMIM #272700) refers to “Tauro” (Bull) and “dont” (tooth) is a developmental anomaly characterized by a large pulp chamber of a multirooted tooth with an apical displacement of pulp floor and bifurcation of the roots. 70 It may occur as an isolated trait or more often a part of a syndrome. It affects more often permanent dentition than primary teeth. The reported prevalence is 0.5 to 46% and it can occur unilaterally or bilaterally. Usually, molars are affected with increased severity being reported in the second and third molars. 71 Both environmental and genetic factors are responsible for taurodontism. The exact etiology of taurodontism is still unknown, but a delay or failure of Herwig's epithelial root sheath to invaginate into mesenchyme reflects in the apical displacement of root furcation, thus resulting in the development of taurodontism. 72

There are several syndromes where taurodontism has been identified like osteogenesis imperfecta, Torg–Winchester syndrome, TDO, cleft lip, and palate. 73 TDO syndrome is associated with DLX3 gene mutations, which are also associated with amelogenesis imperfecta hypoplastic hypomaturation with taurodontism. 74 Taurodontism is also seen in Torg–Winchester syndrome, which is associated with matrix metalloproteinase 2 (MMP2) genetic mutations. 75

## Conclusion

Tooth developmental anomalies involving size, shape, number, quality, or quantity of tooth structure involve alterations in dentitions, which result in dental disharmony causing functional and aesthetic problems. Multiple genes are known to be engaged in normal tooth development, and the complex interactions between genetic, epigenetic, and environmental factors result in the development of dental anomalies. The identiﬁcation of the genetic causes of these anomalies enables clinicians and researchers for a better understanding of molecular pathogenesis. In the near future, it helps provide a more accurate diagnosis and biological-based treatment for these dental anomalies.
